# Analysis of whole genome sequencing for the *Escherichia coli* O157:H7 typing phages

**DOI:** 10.1186/s12864-015-1470-z

**Published:** 2015-04-08

**Authors:** Lauren A Cowley, Stephen J Beckett, Margo Chase-Topping, Neil Perry, Tim J Dallman, David L Gally, Claire Jenkins

**Affiliations:** Gastrointestinal Bacteria Reference Unit, Public Health England, 61 Colindale Ave, London, NW9 5HT UK; Biosciences, College of Life and Environmental Sciences, University of Exeter, Laver Building, North Park Road, Exeter, EX4 4QE UK; Division of Immunity and Infection, The Roslin Institute, R(D)VS, University of Edinburgh, Edinburgh, EH25 9RG UK

## Abstract

**Background:**

Shiga toxin producing *Escherichia coli* O157 can cause severe bloody diarrhea and haemolytic uraemic syndrome. Phage typing of *E. coli* O157 facilitates public health surveillance and outbreak investigations, certain phage types are more likely to occupy specific niches and are associated with specific age groups and disease severity. The aim of this study was to analyse the genome sequences of 16 (fourteen T4 and two T7) *E. coli* O157 typing phages and to determine the genes responsible for the subtle differences in phage type profiles.

**Results:**

The typing phages were sequenced using paired-end Illumina sequencing at The Genome Analysis Centre and the Animal Health and Veterinary Laboratories Agency and bioinformatics programs including Velvet, Brig and Easyfig were used to analyse them. A two-way Euclidian cluster analysis highlighted the associations between groups of phage types and typing phages. The analysis showed that the T7 typing phages (9 and 10) differed by only three genes and that the T4 typing phages formed three distinct groups of similar genomic sequences: Group 1 (1, 8, 11, 12 and 15, 16), Group 2 (3, 6, 7 and 13) and Group 3 (2, 4, 5 and 14). The *E. coli* O157 phage typing scheme exhibited a significantly modular network linked to the genetic similarity of each group showing that these groups are specialised to infect a subset of phage types.

**Conclusion:**

Sequencing the typing phage has enabled us to identify the variable genes within each group and to determine how this corresponds to changes in phage type.

**Electronic supplementary material:**

The online version of this article (doi:10.1186/s12864-015-1470-z) contains supplementary material, which is available to authorized users.

## Background

*Escherichia coli* O157:H7 is the most prevalent Shiga toxin producing *E. coli* (STEC) serotype in the UK and has the most severe impact on human health [1]. STEC O157 symptoms can range from mild gastroenteritis to severe bloody diarrhoea and in more extreme cases haemolytic uraemic syndrome (HUS) [2]. The very young, elderly and immune-compromised are particularly at risk of HUS. A recent Public Health England (PHE) study found incidence to be as high as 1.78 per 100,000 person-years with up to 33% of cases being hospitalised (Gastrointestinal Bacterial Reference Unit (GBRU) in house data). The GBRU at PHE receives approximately 1000 STEC O157 samples per year. Recent outbreaks in the UK have been foodborne or linked to petting farms [3-5]. For purposes of public health surveillance and outbreak investigations, STEC strains are differentiated by phage typing and multilocus variable number tandem repeat analysis [6].

Bacteriophages are viruses that infect bacteria and cause bacterial lysis and cell death, but can also promote horizontal gene transfer between bacteria, play an important role in dynamic bacterial genome evolution and can regulate the abundance and diversity of bacterial communities through co-evolution [7]. There are a range of phages that infect *Escherichia coli* that progress either to a lytic or lysogenic phase after infection. A lytic phase will cause cell lysis whereas in lysogenic phase the phage becomes integrated into the host genome and becomes a prophage. Prophages are important as they often encode additional factors not directly linked to phage production that may provide an evolutionary advantage to the bacterial host enabling survival of the embedded prophage. These include factors that promote colonisation of animal hosts as well as their regulators [8,9]. Bacteriophage specificity is, in part, dependent on the ability of tail fiber proteins to bind to specific receptors on the bacterial host [10].

Phage-typing of STEC O157 is a scheme based on the use of 16 bacteriophages that produce a phage infection profile for a strain based on the level of lysis achieved by each phage [11] and has been used to categorize outbreaks and sporadic cases. Today 80% of all STEC O157 strains typed are PT 8, 21/28, 2, 4 or 32 in the UK (GBRU in house data). Certain PTs are more likely to be associated with human infection and so far there is little understanding of the basis for this. While ongoing work is focused on sequencing and analysis of the bacterial strains, we propose that further insight into relevant strain differences can be gained by also understanding the typing phages themselves and the basis of their infection selectivity. A longer term aim of the work is to understand the factors that mediate resistance and susceptibility in the phage-bacterium relationship.

Little is known about the molecular basis for the interaction between phages and different strains of different phage types, however we can interrogate the phage infection profile of who-infects-whom as a bipartite (two-mode) network. Two common methods for analysing community structure in bipartite data are nestedness and modularity. Nestedness is a way of measuring the ranges of both host resistance and phage infectivity across a specialist to generalist gradient. Specialists are assumed to have strategies that are subsets of those which are more generalised. Modularity is the degree to which a network can be split into distinct modular groupings of phage and bacteria such that there are many infections within rather than between groups [12].

The 16 phages in the STEC phage-typing scheme are made up of 14 T4 phages and 2 T7 phages. An example of a T7 phage has been sequenced previously and T7 are known to consist of a single ‘chromosome’ carrying about 30 genes [13]. The 5’ end genes of the chromosome are expressed at an early stage of infection and their products are involved in the induction of host RNA polymerase for transcription and control the expression of other phage genes in a positive feedback mechanism. Genes that are expressed later are involved in the metabolism of phage DNA and code for capsid proteins or are involved in the assembly of infective progeny particles [13]. T4 phages have much larger genomes with 300 putative genes, only 62 of these have been found to be ‘essential’ under laboratory conditions [14]. The order of expression works in a similar way to T7 phage.

The STEC O157 typing phages 5, 7 and 10 from the typing scheme have previously been sequenced [15-17]. Our sequencing results are consistent with previously published sequences. We build on this data by placing the previously sequenced phages into similarity groups within the typing phages. The aim of this study was to analyse the genome sequences of 16 (fourteen T4 and two T7) STEC O157 typing phages (TPs) and to identify genes that may account for differences in infectivity between related phages.

## Methods

### Phage propagation and DNA extraction

**Figure 1:**
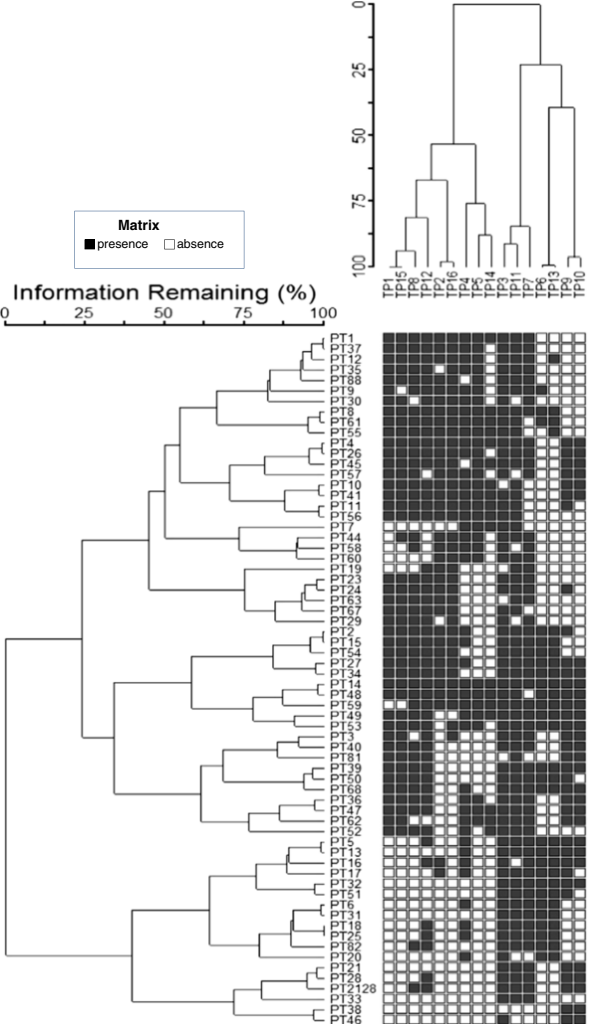
**Two-way cluster analysis dendrogram of 66 phage types and 16 typing phages.** The matrix of shaded squares represents the phage type × typing phage matrix, while the dendrograms show the clustering. The dendrograms are scaled by Wishart ‘s (1969) objective function, expressed as the percentage of information remaining at each level of grouping (McCune and Grace, 2002). Each square represents the presence (black) and absence (white) of a reaction with a given typing phage. The three phage type clusters and the 4 typing phage clusters are indicated at the node with numbers.

The typing phages were obtained as a gift from the National Microbiology Laboratory, Winnipeg, MN, Canada to GBRU in the late 1980s. To propagate the phage, 0.1 ml of the propagating strain (Additional file 1: Table S1, Figure 1) was inoculated into 2 × 20 ml of single strength Difco nutrient broth and 0.1 ml of test phage was added to one and the other kept as a control. The bottles were incubated and turbidity was monitored. When lysis was judged to be at its maximum compared to the control, a small amount of the phage solution was centrifuged at 2,200 g for 20 min. The supernatant was removed and spotted onto a flooded plate of propagating strain as a test; the plate was dried and incubated at 37°C overnight. The plates were examined for lysis and if positive the phage lysate was sterilized by filtration and stored at 4°C.

All phages were filtered before extraction took place. Eleven (phages 1, 3, 4, 5, 6, 7, 8, 9, 12, 13 and 14) of the 16 phages were extracted using the QIAamp UltraSens Virus kit (Qiagen, UK) following the manufacturer’s instructions. This method failed to produce a high enough concentration of DNA for the remaining phages (2, 10, 11, 15 and 16) and these were extracted using a Zinc Chloride protocol [18]. Briefly, 20 μl of a 2 M Zinc chloride solution was added to 1 ml of sample and incubated for 5 min at 37°C. The sample was then centrifuged at 10000 rpm and the supernatant was removed. The pellet was resuspended in 500 μl of TES buffer (0.1 M Tris–HCl, pH8; 0.1 M EDTA and 0.3% SDS) and then incubated at 60°C for 15 min. Subsequently, 60 μl of a 3 M potassium acetate solution was added and the sample left on ice for 10 to 15 min. Following the formation of a white, dense precipitation the sample was centrifuged for 1 min at 12000 rpm and the supernatant removed to a new tube. To this an equal volume of isopropanol was added, the solution vortexed and left on ice for 5 min. The solution was centrifuged and evaporated simultaneously using a Speedy-Vac machine and the pellet washed with 70% ethanol before being resuspended in 20–100 μl TE (10 mM Tris–HCl, pH8; ImM EDTA). Samples were pooled by five extractions to give a higher yield of DNA. This method also failed to produce high enough concentration of DNA for sequencing TP 2 and 16 and we were ultimately unable to obtain sequencing data for these two TPs.

#### Sequencing

The first set of phages (1, 3, 4, 5, 6, 7, 8, 9, 12, 13 and 14) was sequenced at The Genome Analysis Centre (TGAC) on an Illumina MiSeq. Illumina TruSeq DNA library construction was performed and sequencing of the libraries was pooled on one run using 150 bp paired-end reads, this generated greater than 1 Gbp of data for the run. Data was then quality controlled, basecalling was performed and it was formatted. The second set of phages (10, 11 and 15) was sequenced at the Animal Health and Veterinary Laboratories Agency on an Illumina GAII. The library construction was performed using a Nextera DNA sample preparation kit (Illumina) and then sequenced in the same manner as the other set.

#### Bioinformatic sequencing analysis

Reads for all phages apart from TP 15 were *de novo* assembled into whole genomes using Velvet optimizer with a range of k-mer values from 90–120 [19] and annotated using Prokka 1.5.2 and output as GenBank files [20]. The genomes were visualised in the multiple genome alignment tool Mauve with a progressive alignment to visualise similarities and differences between them based on sequence content. The reads assembled into between 1 and 7 contigs for each phage.

TP15 could not be assembled correctly because the propagation process had induced other temperate phages in the genome of the propagating strain and the DNA had been co-extracted. Subsampling to x150 coverage and the genome assembler SPAdes with a better low frequency k-mer elimination step [21] was used to overcome this issue and resolve 15 true typing phage 15 contigs from the assemblies. The sequencing data has been made publicly available in the Short Read Archive under study alias PRJNA252693 and Genbank accession numbers for each phage can be found in the availability of supporting data section.

#### Euclidian tree

Data from PHE on the protocol used to identify phage types (Additional file 1: Table S3, Additional file 1: Table S2) was converted into binary (presence/absence) format. In the original scheme there were 66 established phage types (PT) and 16 typing phages (TP). This set of data was analysed using a two-way cluster hierarchical agglomerative analysis in PC-ORD software version 6.08 (MJM software Design, Gleneden Beach, OR). The clustering was performed with Euclidian distance matrix and Ward linkage method.

The optimal number of groups of plots was first evaluated with multiresponse permutation procedure, seeking the solution with fewest number of groups but the greatest gain in *A-*statistics [22].

#### Modularity and nestedness

Modularity of the network was calculated using the LPAb + algorithm [23] which uses label propagation coupled with greedy multistep agglomeration to identify the communities (made of members of both types of nodes (bacteria and phage)) that maximise modularity in bipartite networks. As LPAb + is stochastic we choose the best modularity score, Q_B_, returned from 1,000 trials each time we use the algorithm. Code for performing the modularity analysis is supplied [24].

Nestedness statistics were calculated using FALCON [25]. The nestedness measures used were NODF [26], NTC [27,28] and BR, the discrepancy score of Brualdi and Sanderson, 1999 [29]. NODF and NTC scores take values in the range [0,100], whilst BR is the absolute number of differences between the input and a maximally packed matrix. NODF has been recalculated here as NODF = 100-NODF, so that lower measure scores show greater nestedness with 0 representing perfect nestedness for each of the measures.

We tested for significance of both modularity and the nestedness found in our phage-bacteria infection network using two null models based on properties of our network. Null model one is a Bernoulli random null model where connections between phage j and bacteria i are made with probability *p*_*ij*_ *= F/M*, where F is the total number of edges in our network (number of infecting interactions) and M is the maximum number of potential interactions (number of TP’s × number of PT’s). Null model two is based on the information in the rows and columns in the network [30]; where a connection between phage *j* and bacteria *i* is made with probability *p*_*ij*_ *= 0.5 (d*_*j*_*/r + k*_*i*_*/c)* where *d*_*j*_ is the number of infections caused by phage *j*, *r* is the number of PTs, *k*_*i*_ is the number of phage that can infect bacteria *i* and *c* is the number of TPs. We tested 1,000 null matrices against our network for each null model in the modularity analysis, whilst we used the adaptive ensemble of FALCON for nestedness analysis and report the ensemble size used (N), p-values (probability of finding a more modular/nested network from the null model) and z-scores (effect size; the number of standard deviations our network was away from the mean average found in each null model).

#### BRIG plot

BRIG (Blast Ring Image Generator), a genome comparison tool [31], was used to compare similarities between the 12 T4 like typing phages by inputting all of the GenBank files for the assembled genomes and plotting blast hits against a MultiFASTA file of all of the phages. The image was displayed as a series of concentric rings with the central ring being the MultiFASTA reference; each outer ring displays hits (i.e. genomic regions that show a high percentage similarity to the central reference genome) for each phage. BRIG was also used to show the comparison of phages 9 and 10 (the two T7 like typing phages) against phage 9 as a reference.

#### SeqFindR and Easyfig plots

SeqFindR, a bioinformatics tool developed by the Beatson Laboratory at the University of Queensland, was used to identify gene presence and absence in the phage genomes. Easyfig [32] was used to visualise the coding regions and colour the accessory genes in red for each phage group.

#### Tail fiber analysis

Tail fiber encoding genes were extracted from the GenBank files of the typing phages and the protein sequences aligned using MEGA 5.2. The alignment told us how many changes in protein sequence there were within the groups.

## Results

**Figure 2:**
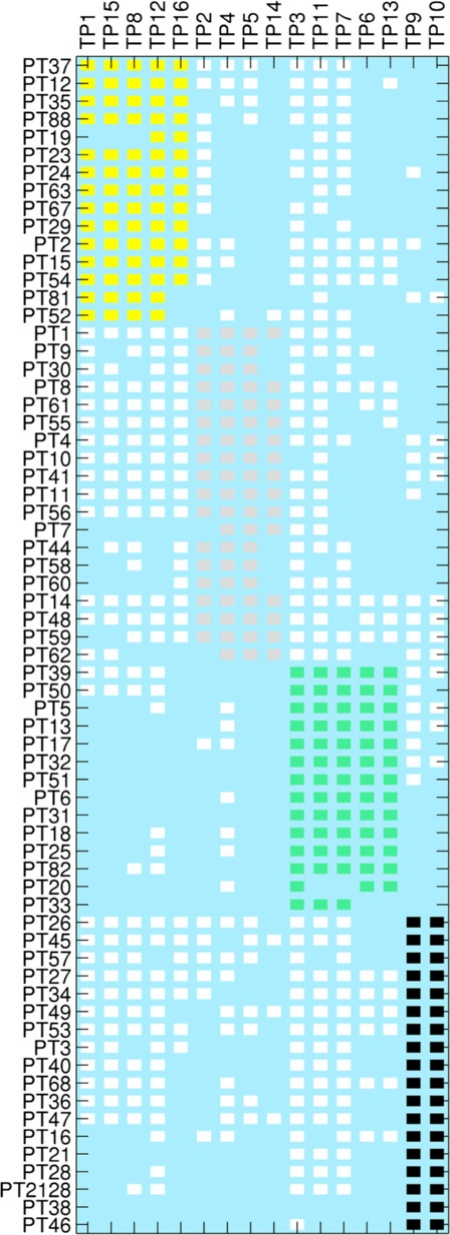
**A visual representation of the modularity seen within the system with modules coloured.** Phage type (PT) is represented on the y axis and Typing phage (TP) is represented on the x axis and the matrix showing presence of a reaction with that phage as a white or coloured block. The 4 observed modules are coloured as yellow, pink, green and black.

In the phage typing scheme there are 14 T4-like bacteriophages (TP1-8 and TP11-16) and two T7-like bacteriophages (TP9 and TP10). The reactivity of each of the typing phages with respect to the STEC O157 phage typing scheme was analysed. The two-way Euclidian cluster analysis combined the independent clustering of 66 STEC O157 bacterial phage types and the 16 typing phages into a single diagram and highlighted the associations between groups of phage types and typing phages (Figure 1). The analysis showed that the STEC O157 phage typing scheme formed a weak (Q_b_ = 0.1575 (Table 1)) but significantly modular network where the TP groups were each specialised to infect a subset of PTs (Figure 2). There also exists a large number of between module interactions. Furthermore, the majority of PTs of STEC O157 react with at least one member of each group of typing phages. These groups can be regarded as universally infective against STEC O157. Using statistical tests we also found that the nestedness of our interaction network was statistically significantly different from that found under randomly formed networks (Table 1). This indicates a correlation between phage infectivity range and the resistance range of the host. These phages have been designed and chosen to infect STEC O157 and create a typing scheme with the simplest and minimum selection of phages so it makes sense that the system is nested.

**Table 1:** **Summary statistics for nestedness and modularity analysis**

		**Modularity**	**Nestedness**		
Measure		Q_B_	NODF	NTC	BR
Measure score	x	0.1575	27.9199	30.2532	130
Null model 1	N	1000	1300	1300	1300
	p-value	<1/N	<1/N	<1/N	<1/N
	z-score	4.8602	-7.5382	-11.9831	-11.7632
Null model 2	N	1000	1000	1000	1000
	p-value	<1/N	<1/N	<1/N	<1/N
	z-score	5.7693	-4.6740	-6.7842	-7.1554

Barber’s modularity (Q_b_) and three nestedness measures (NODF, NTC and BR) were calculated. Two null models were used to generate ensembles of networks (of size N) to evaluate the strength of the modularity and nestedness observed in the classified *Escherichia coli* O157:H7 phage-bacteria infection network. This is done by reporting the significance (as a *p*-value) and effect size (as a z-score) of the phage-bacteria infection network relative to the networks found in each null model ensemble. Note that, due to differences in how these measures are calculated, for modularity a positive z-score indicates that modularity is greater in the observed network than the mean average of the ensemble; whilst in the nestedness analysis a negative z-score indicates the observed network is more nested than the mean nestedness found within the null ensemble. The classified *Escherichia coli* O157:H7 phage-bacteria infection network was found to be both more nested and more modular than any of the networks generated by the tested null models.

Fourteen of the 16 phages in the typing scheme were sequenced and successfully assembled. Despite several attempts, sequencing of typing phages 2 and 16 failed due to insufficient quantities of DNA extracted from the phage propagation preparations.

**Figure 3:**
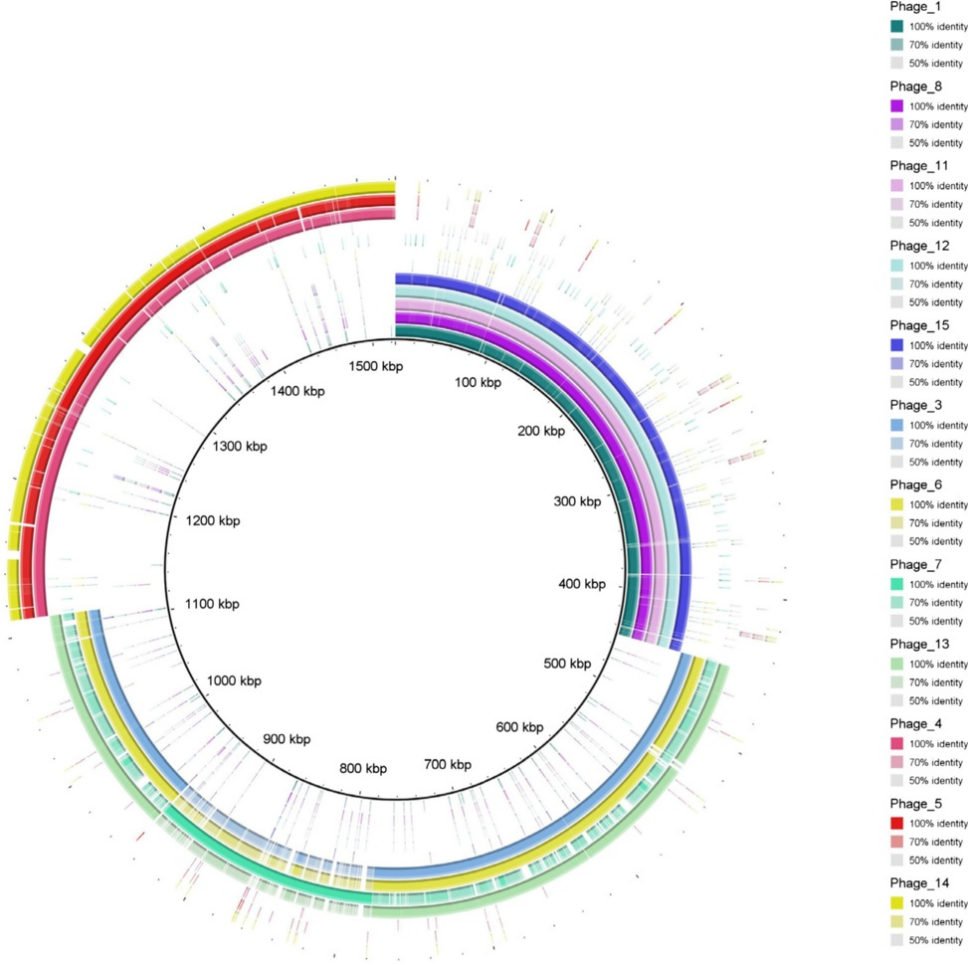
**A genomic representative diagram drawn with BRIG of T4-like phage similarities, the coloured regions indicate high pairwise genomic sequence similarity according to blastn.** Legend indicates which colours correspond with which phages and the shade of that colour indicates what level of similarity is observed. Central ring is multifasta of all T4-like phage genomes and each consecutive ring represents the similarity with a single phage. The multifasta and rings are in the same phage order.

The BRIG plot showed that the 12 sequenced T4-like bacteriophages formed three distinct groups of similar genomic sequences (Figure 3). Group 1 included typing phages 1, 8, 11, 12 and 15; Group 2 comprised typing phages 3, 6, 7 and 13 and typing phages 4, 5 and 14 were in Group 3. Although the sequencing for TP2 and TP16 failed, the modularity analysis indicates that TP16 belonged to Group 1 and TP2 belonged to Group 3 (Figure 2). The TPs varied significantly in size between the three groups: the members of Group 1 were 93,000–95,000 bp, Group 2 members were 165,000–175,000 bp and those in Group 3 were 135,000–140,000 bp.

**Figure 4:**
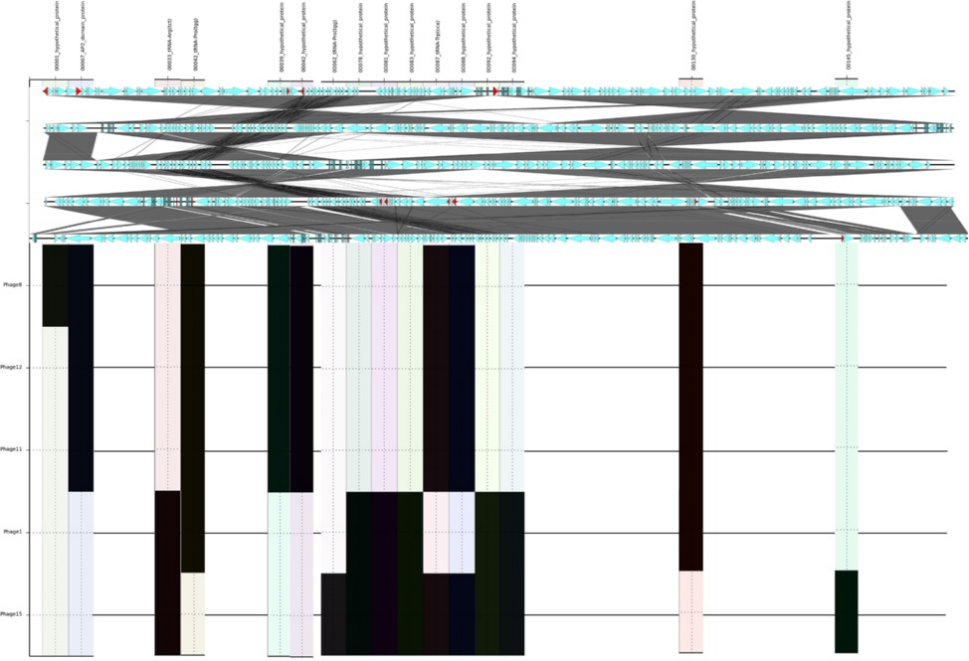
**SeqFindR and Easyfig image combined representing the accessory gene content of group 1.** Genomes of each phage in group 1 are represented by the Easyfig image showing linear visualisation of the genome and coding regions represented by arrows, accessory genes are coloured red. The order of phage genomes in the linear visualisation and the accessory content blocking is 8, 12, 11, 1 and 15 and was chosen based on similarity clustering in SeqFindR. Hits for the accessory genes in each genome are represented in labelled columns in the SeqFindR image underneath each accessory gene.

The Group 1 phages (TP1, 8, 11, 12 and 15) were approximately 90,000 bp in length. These five phages were highly similar in genetic sequence content. The location, annotation and presence of accessory genes within Group 1 are shown in Figure 4, Additional file 1: Table S3. Figure 4 shows that there were 6 genes found in TP1 but absent in TP8, 11, 12 and 15 (five were annotated as hypothetical proteins and one tRNA). There were also five genes present in TP8, 11, 12 and 15 but not in TP1 (three were annotated as hypothetical proteins, one as AP2 domain protein and one was a tRNA gene) (Figure 4, Additional file 1: Table S3). TP8 was missing a region annotated as a putative prophage that was present in the other members of the group. With the exception of TP11, the Group 1 TPs are most closely related to each other by the two-way Euclidian cluster analysis demonstrating the link between gene content and phage typing profile.

**Figure 5:**
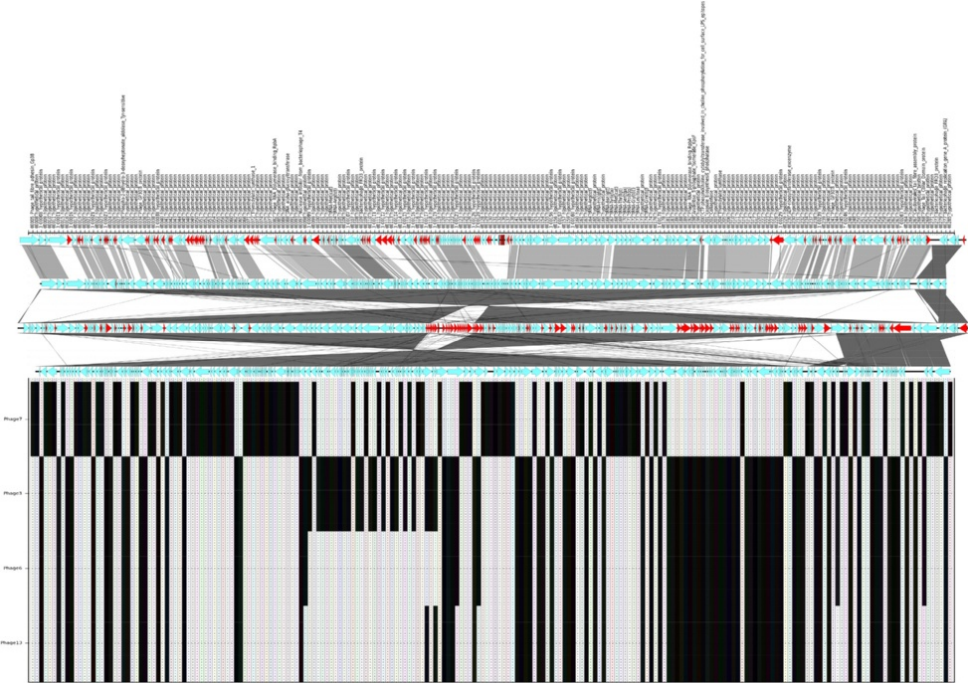
**SeqFindR and Easyfig image combined representing the accessory gene content of group 2.** Genomes of each phage in group 2 are represented by the Easyfig image showing linear visualisation of the genome and coding regions represented by arrows, accessory genes are coloured red. The order of phage genomes in the linear visualisation and the accessory content blocking is 7, 3, 6 and 13 and was chosen based on similarity clustering in SeqFindR. Hits for the accessory genes in each genome are represented in labelled columns in the SeqFindR image underneath each accessory gene.

The typing phages in Group 2 (TP 3, 6, 7, and 13) were between 160–170,000 bp in length. The genomes were almost twice the size of the phages in Group 1 and exhibited less similarity. The accessory genes found in Group 2 were mostly annotated as encoding hypothetical proteins (Figure 5, Additional file 1: Table S4). The two-way Euclidian cluster analysis highlighted a close relationship between TP6 and TP13 and this corresponded with the level of sequence similarity of these two typing phages illustrated in Figure 5.

**Figure 6:**
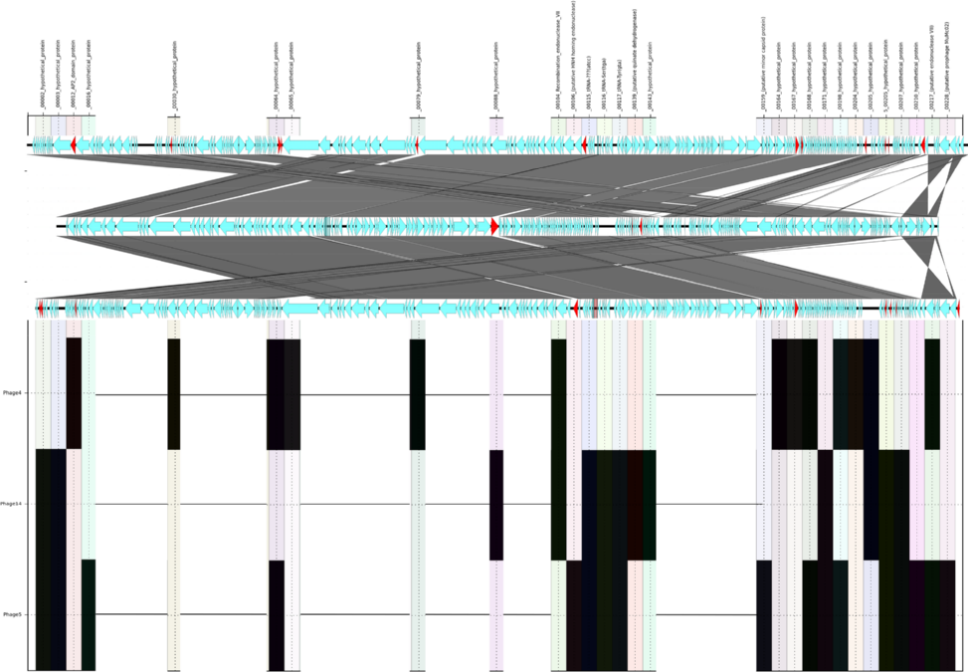
**SeqFindR and Easyfig image combined representing the accessory gene content of group 3.** Genomes of each phage in group 3 are represented by the Easyfig image showing linear visualisation of the genome and coding regions represented by arrows, accessory genes are coloured red. The order of phage genomes in the linear visualisation and the accessory content blocking is 4, 14 and 5 and was chosen based on similarity clustering in SeqFindR . Hits for the accessory genes in each genome are represented in labelled columns in the SeqFindR image underneath each accessory gene.

Typing phages 4, 5 and 14 were designated Group 3 and were 130–140,000 bp in length. Figure 6 shows the location, annotation and presence of accessory genes within Group 3. Figure 6 demonstrates that there were 29 gene differences within the group and the majorities (19) were annotated as hypothetical proteins. In addition, three genes encoded putative endonucleases and there were three genes designated tRNAs (Figure 6, Additional file 1: Table S5). The typing phages in Group 3 were most closely related to each other by the two-way Euclidian cluster analysis (Figure 1).

**Figure 7:**
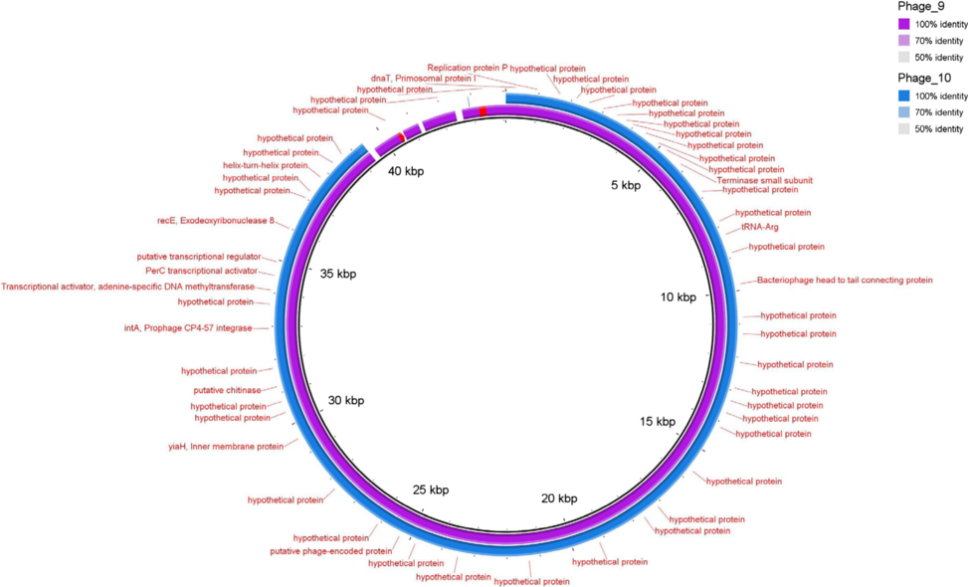
**A genomic representative diagram drawn with BRIG, the coloured regions indicate high pairwise genomic sequence similarity according to blastn.** The legend indicates which colours correspond with which phages. The central ring is a genbank file of Phage 9 as a reference and annotations of genes in red. The first ring is representative of Phage 9 and the second ring is representative of Phage 10 and the shade indicates the level of genomic similarity observed.

Phages 9 and 10, the two Podoviridae or T7 like phages that are found in the typing scheme, were successfully sequenced, assembled and annotated and revealed 40–45000 bp genomes consistent with the published sequences of T7 bacteriophages (Figure 7). Phages 9 and 10 differed by only three genes (annotated as encoded hypothetical proteins) that were found in Phage 9 but not in phage 10. The two-way Euclidian cluster analysis confirmed the close relationship between TP9 and TP 10 in terms of phage type profile. It also showed that there were six STEC O157 phage types (PT 2, 11, 17, 24, 50, and 51) that react with TP9 but not TP10 and none of the phage types react with TP10 but not TP9 (Figure 1). These three hypothetical proteins could be the key to the differences in the reactivity profiles of TP9 and 10.

Tail-fiber encoding genes were analysed within each group and it was found that there were changes in the amino acid sequence for certain members of each group. Within the group 1 typing phages, phages 1 and 15 had 3 changes in amino acid sequence in their tail fibers, 2 of which were shared and 1 each unique to each phage. Within the group 2 typing phages, phage 7 has 47 changes in its amino acid sequence and 3 amino acid deletions. Within the group 3 typing phages, the same single position in all 3 members of the group has a different amino acid present and additionally there was another single position in typing phage 14 that had a different amino acid. The T7-like phages had identical tail fiber genes. There was no genetic similarity between tail fiber genes found in different groups.

## Discussion

Phage-host interactions are key to understanding the virulence and success of *E. coli* O157 but little is known about the typing phages used in the O157 typing scheme. Sequencing these phages has enabled us to group the T4-like Myoviridae and the two Podoviridae or T7-like phages members of the typing phage scheme into four groups based on their sequence similarity. The two-way Euclidian cluster analysis demonstrated that similar phage groups react with STEC O157 in a similar way with closely related reaction profiles. The sequencing data also highlighted that a small number of gene differences may be responsible for the subtle differences in reaction profiles within the groups.

The large proportion of genes annotated as encoding hypothetical proteins hindered our investigations into the mechanisms of host-phage interactions. Attempts were made to annotate these genes further using protein BLAST and HMMER but only uncharacterised proteins were hit. However, the determination of which genes vary within each group will enable us to focus on the genes that may play a key role in the mechanisms of interactions between specific typing phages and strains belonging to specific phage types. For example, in Group 1, there were five genes that were found only in TP8, 11 and 12 and three PTs (PT21/28, 59 and 82) that only react with these TPs. The proteins encoded by these five genes may play a key role in the host-phage mechanisms between TP8, 11 and 12 and strains of STEC O157 belonging to PT21/28, 59 and 82. PT21/28 is the most common PT in the UK and is significantly associated with HUS [33]. Further details of unique host-phage interactions are described in Additional file 1: Table S6 and the genes referred to within can be found in Figures 4, 5 and 6.

Analysis of tail fibers genes showed that typing phages 1, 15, 7 and each individual member of Group 3 had different protein sequences encoded to the other members of their group. The changes that were found could partially account for infectivity differences [34]. These could explain a few of the differences in host specificity seen within those groups, although this will not apply to the T7-like typing phages that have had identical predicted tail fiber proteins.

Certain typing phages had almost identical genomes but different host susceptibility profiles, for example, TP11 belonged to the Group 1 phages but had a similar host susceptibility profile to the Group 2 phages. Each phage in the typing scheme has its own propagating strain (see Additional file 1: Figure S1, Table 1) so it is also possible that host-induced modification occurs [35]. For example, the propagating strains for the closely related typing phages TP9 and TP10 are STEC O157 PT2 and PT32, respectively. Modifications may be a result of methylation or other phenotypic changes that are not evident in the genome but may affect the host range of the virus.

Phenotypic differences in susceptibility patterns in genetically similar phages could be explained by the transcription order of genetic loci in the phage genome. It has been suggested that gene synteny constrains adaptation and is important for fitness and, therefore, infectivity of bacteriophages [36]. The order of transcription may be important in overcoming the host response to infection. The phages that transcribe their genetic loci in a different order may be killed and degraded by the host response, for example, TP 8, 11 and 12 are almost identical but have a different gene order and this may be key to their different infection profiles.

Our analysis showed that the significantly modular network exhibited by the STEC O157 phage typing scheme was linked to the genetic similarity groups mentioned above showing that these groups are specialised to infect a subset of PTs. However, the typing scheme as a whole is also significantly nested; more generalised phages minimise the number of phages needed in the scheme. Both of these network structures have also been found in other phage-bacteria infection networks [37,38]. The most common PTs in the UK: 2, 8, 21/28 and 32 are all found in different modules, meaning there is an abundant PT in each module. When looking at these PTs with nestedness, PT 8 and 2 both have a phage susceptibility range of 14 and 13 respectively so are quite generalised but PT 21/28 and 32 both have a host range of 7, and lie more towards the specialised end of the spectrum. It is interesting that the more abundant strains seem to appear at two levels of host range – perhaps suggestive of a trade-off between host range and phage productivity. It would be interesting to see, in conditions where the phages are allowed to evolve with their hosts, if a more modular network arises with further specialisation of the phages to maintain a kill-the-winner dynamic and less broad range infectivity [39]. This is an artificial system that we are observing and it is likely that we would see a different network arising in nature’s ecological systems.

Phage-typing has been used for epidemiological and surveillance studies by a number of groups [40,41] for different organisms. Phage-type association with increased strain virulence is of high interest to public health workers dealing with STEC O157, the replacement of phage-typing with whole genome sequencing should still incorporate our knowledge of phage type and associated virulence. For this reason it is valuable to find the molecular markers associated with high frequency and highly pathogenic phage types; elucidating the determinants underpinning differences in phage typing should contribute to this.

Phage-mediated therapies will continue to be an area of interest as we struggle with resistance to conventional antibiotics. It makes sense that moving forward there will be considerable interest in being able to predict bacterial susceptibility to ‘treatment’ phages based on sequence information alone. Furthermore, the next step would be modification of specific phages to improve their targeting/activity. This will rely on understanding of the phage genes that govern the specificity of infection in different backgrounds. The place to start is with certain key bacterial pathogens and a bank of phages.

## Conclusions

In this study, the STEC O157 typing phages we clustered into four distinct groups of similar genomic sequences, that broadly correlated with phage typing profile groups determined by two-way Euclidian clustering. Genetic variation within the TP groups may explain the subtle differences between the phage typing profiles exhibited by the *E. coli* O157 typing phages. This analysis was hindered by the lack of detailed annotation of protein encoding genes in T4 and T7-like phages. The impact of the order of transcription of the blocks of genetic loci and the role of host-induced modification further confound the analysis. However, sequencing the typing phage has enabled us to identify the variable genes within each group and to determine how these correspond to changes in phage type. Future studies will focus on the genes that appear to alter host-phage interactions and we aim to identify bacterial genes that influence typing phage resistance and susceptibility using random mutagenesis approaches. In order to understand the best combination of strains and individual phages to work with, the network of interactions needs to be analysed. This information can also provide insight on how phage typing can potentially be simplified in the future. A better understanding of the genetic differences between bacterial phage types, and the possible differences in virulence factors, could help elucidate why different phage types occupy specific niches and are associated with different patient age groups and disease severity.

## Availability of supporting data

The raw sequencing reads have been deposited in the short read archive under project alias PRJNA252693. The assembled sequences and annotations of the typing phages have been deposited in Genbank under the following accessions;

Phage 1: KP869100

Phage 3: KP869101

Phage 4: KP869102

Phage 5: KP869103

Phage 6: KP869104

Phage 7: KP869105

Phage 8: KP869106

Phage 9: KP869107

Phage 10: KP869108

Phage 11: KP869109

Phage 12: KP869110

Phage 13: KP869111

Phage 14: KP869112

Phage 15: KP869113

All other supporting data is included as additional files.

## Additional file

Additional file 1: Table S1. Propagating strain table. Table showing propagating strain and corresponding typing phage number that the strain propagates. **Table S2.** *E. coli* O157 phage typing scheme. Table showing reactions of the *E. coli* O157 type strains with the typing phages. **Table S3.** Table of the accessory variation of the Group 1 typing phages. Table detailing the accessory variation of Group 1 as depicted in figure 4. **Table S4.** Table of the accessory variation of the Group 2 typing phages. Table detailing the accessory variation of Group 2 as depicted in figure 5. **Table S5.** Table of the accessory variation of the Group 3 typing phages. Table detailing the accessory variation of Group 3 as depicted in figure 6. **Table S6.** Table of unique reactions. Table representing unique reaction that only occur within a subset of groups 1, 2 and 3 with specific PTs and number of genes found only in that subset. **Figure S1.** Phylogenetic tree of propagating strains. Phylogenetic tree of propagating strains for each typing phage and sakai as a reference. **Figure S2.** Visual representation of nestedness. A visual representation of the degree of nestedness found within the classified *E. coli* O157 phage-bacteria infection network. **Figure S3.** Electron microscopy image of typing phage 7 A representation of the the T4-like long-tailed phage morphology within the typing phages. **Figure S4.** Electron microscopy image of typing phage 9 A representation of the T7-like short-tailed phage morphology within the typing phages.

## Abbreviations

STEC: Shiga Toxin producing *Escherichia coli*
HUS: Haemolytic uraemic syndrome
PT: Phage-type
TP: Typing phage
TGAC: The genome analysis centre
AHVLA: Animal health and veterinary laboratories agency
PHE: Public health England
GBRU: Gastrointestinal bacterial reference unit
BRIG: Blast ring image generator
