# Supplementary material for: Analysis of whole genome sequencing for the Escherichia coli O157:H7 typing phages
Source: BMC Genomics. 2015 Apr 8;16(1):271. doi: 10.1186/s12864-015-1470-z (PMC4429339; doi:10.1186/s12864-015-1470-z)
Supplement: Additional file 1: Table S1. — Propagating strain table. Table showing propagating strain and corresponding typing phage number that the strain propagates. Table S2. E. coli O157 phage typing scheme. Table showing reactions of the E. coli O157 type strains with the typing phages. Table S3. Table of the accessory variation of the Group 1 typing phages. Table detailing the accessory variation of Group 1 as depicted in figure 4. Table S4. Table of the accessory variation of the Group 2 typing phages. Table detailing the accessory variation of Group 2 as depicted in figure 5. Table S5. Table of the accessory variation of the Group 3 typing phages. Table detailing the accessory variation of Group 3 as depicted in figure 6. Table S6. Table of unique reactions. Table representing unique reaction that only occur within a subset of groups 1, 2 and 3 with specific PTs and number of genes found only in that subset. Figure S1. Phylogenetic tree of propagating strains. Phylogenetic tree of propagating strains for each typing phage and sakai as a reference. Figure S2. Visual representation of nestedness. A visual representation of the degree of nestedness found within the classified E. coli O157 phage-bacteria infection network. Figure S3. Electron microscopy image of typing phage 7 A representation of the the T4-like long-tailed phage morphology within the typing phages. Figure S4. Electron microscopy image of typing phage 9 A representation of the T7-like short-tailed phage morphology within the typing phages. [file 12864_2015_1470_MOESM1_ESM.doc]

**Additional files**

**Table 1.** Table showing propagating strain and corresponding typing phage number that the strain propagates in the first column. The second column tells us what PT each propagating strain is.

| Propagating strain for typing phage number | Phage type |
| --- | --- |
| PS 1 | PT4 |
| PS 2 | PT4 |
| PS 3 | PT21/28 |
| PS 4 | PT14 |
| PS 5 | PT14 |
| PS 6 | PT14 |
| PS 7 | PT2 |
| PS 8 | PT14 |
| PS 9 | PT2 |
| PS 10 | PT32 |
| PS 11 | PT2 |
| PS 12 | PT14 |
| PS 13 | PT2 |
| PS 14 | PT14 |
| PS 15 | PT2 |
| PS 16 | PT4 |

**Table 2.** Reactions of the *E. coli* O157 type strains with the typing phages at routine test dilution, this data was used to construct the Euclidan tree shown in Figure 1.

| **Phage Type** | **1** | **2** | **3** | **4** | **5** | **6** | **7** | **8** | **9** | **10** | **11** | **12** | **13** | **14** | **15** | **16** |
| --- | --- | --- | --- | --- | --- | --- | --- | --- | --- | --- | --- | --- | --- | --- | --- | --- |
| **1** | **<CL** | **CL** | **SCL** | **SCL** | **SCL** | **-** | **<SCL** | **CL** | **-** | **-** | **CL** | **CL** | **-** | **<SCL** | **CL** | **SCL** |
| **2** | **CL** | **CL** | **<CL** | **<<SCL/-** | **-** | **<SCL** | **<SCL** | **CL** | **OL** | **-** | **CL** | **CL** | **CL** | **-** | **CL** | **SCL** |
| **3** | **+++** | **-** | **SCL** | **-** | **-** | **-** | **<SCL** | **-°** | **OL** | **OL** | **SCL** | **CL** | **-** | **-** | **SCL** | **SCL** |
| **4** | **CL** | **CL** | **SCL** | **<<SCL** | **<<SCL** | **-** | **<SCL** | **CL** | **OL** | **OL** | **CL** | **CL** |  | **<SCL** | **CL** | **<CL** |
| **5** | **-** | **-** | **SCL** | **SCL** | **-** | **SCL** | **SCL** | **-** | **OL** | **OL** | **<<OL** | **<OL** | **CL** | **-** | **-** | **-** |
| **6** | **-** | **-** | **SCL** | **SCL** | **-** | **+++** | **SCL** | **-** | **-** | **-** | **SCL** | **-** | **SCL** | **-** | **-** | **-** |
| **7** | **-** | **-** | **SCL** | **SCL** | **SCL** | **-** | **-** | **-** | **-** | **-** | **SCL** | **-** | **-** | **SCL** | **-** | **-** |
| **8** | **CL** | **CL** | **<CL** | **<<SCL** | **SCL** | **SCL** | **SCL** | **CL** | **-** | **-** | **CL** | **CL** | **CL** | **<SCL** | **CL** | **<SCL** |
| **9** | **SCL** | **SCL** | **SCL** | **CL** | **SCL** | **+++** | **SCL** | **SCL** | **-** | **-** | **SCL** | **CL** | **-** | **-** | **-** | **SCL** |
| **10** | **SCL** | **SCL** | **-** | **SCL** | **SCL** | **-** | **-** | **SCL** | **<<OL** | **<OL** | **+++** | **CL** | **-** | **CL** | **CL** | **CL** |
| **11** | **SCL** | **SCL** | **SCL** | **SCL** | **SCL** | **-** | **-** | **SCL** | **<<OL** | **-** | **SCL** | **SCL** | **-** | **SCL** | **SCL** | **SCL** |
| **12** | **SCL** | **SCL** | **SCL** | **CL** | **CL** | **-** | **SCL** | **SCL** | **-** | **-** | **+++** | **SCL** | **SCL** | **-** | **SCL** | **SCL** |
| **13** | **-** | **-** | **SCL** | **SCL** | **-** | **+++** | **+++** | **-°** | **<<OL** | **<<OL** | **<<OL** | **-** | **SCL** | **-** | **-** | **-** |
| **14** | **CL** | **CL** | **SCL** | **<<SCL** | **<SCL** | **<SCL** | **<<SCL** | **CL** | **OL** | **OL** | **CL** | **CL** | **CL** | **<<SCL** | **CL** | **<CL** |
| **15** | **SCL** | **+++** | **SCL** | **SCL** | **-** | **+++** | **SCL** | **SCL** | **-** | **-** | **SCL** | **SCL** | **SCL** | **-** | **SCL** | **SCL** |
| **16** | **-** | **+++** | **SCL** | **+++** | **-** | **+++** | **<SCL** | **-** | **<OL** | **<OL** | **-** | **+++** | **SCL** | **-** | **-** | **-** |
| **17** | **-** | **+++** | **+++** | **+++** | **-** | **-/+++** | **SCL** | **-** | **<OL** | **-** | **SCL** | **-** | **+++** | **-** | **-** | **-** |
| **18** | **-** | **-** | **+++** | **+++** | **-** | **-/++** | **+++** | **-** | **-** | **-** | **<<OL** | **-/++** | **<CL** | **-** | **-** | **-** |
| **19** | **-** | **+++** | **-** | **-** | **-** | **-** | **+++** | **-** | **-** | **-** | **<OL** | **+++** | **-** | **-** | **-** | **+++** |
| **20** | **-** | **-** | **+++** | **+++/-** | **-** | **++** | **-** | **-** | **-** | **-** | **-** | **-** | **<SCL** | **-** | **-** | **-** |
| **21** | **-** | **-** | **<<SCL** | **-** | **-** | **-** | **+++** | **-°** | **<OL** | **<OL** | **P -** | **-** | **-** | **-** | **-** | **-** |
| **21/28** | **-** | **-** | **CL** | **-** | **-** | **-** | **SCL** | **+++μ** | **<OL** | **<OL** | **+++μ** | **+++μ** | **-** | **-** | **-** | **-** |
| **22** | **-** | **-** |  | **-** | **-** | **-** |  | **+++** | **<OL** | **<OL** |  | **SCL** | **-** | **-** | **-** | **-** |
| **23** | **<CL** | **CL** | **SCL** | **-** | **-** | **-** | **SCL** | **CL** | **-** | **-** | **CL** | **<CL** | **-** | **-** | **CL** | **SCL** |
| **24** | **CL** | **CL** | **SCL** | **-** | **-** | **-** | **+++** | **CL** | **OL** | **-** | **CL** | **CL** | **-** | **-** | **CL** | **<<SCL** |
| **25** | **-** | **-** | **SCL** | **+++** | **-** | **<SCL** | **<SCL** | **-°** | **-** | **-** | **<<OL** | **<<OL** | **CL** | **-** | **-** | **-** |
| **26** | **<CL** | **SCL** | **+++** | **+++** | **+++** | **-** | **+++** | **CL** | **<OL** | **<OL** | **CL** | **CL** | **-** | **-** | **CL** | **SCL** |
| **27** | **+++** | **<<OL/-** | **SCL** | **+++/-** | **-** | **<<SCL** | **<<SCL** | **CL/-** | **+++** | **+++** | **CL** | **CL** | **CL** | **-** | **SCL** | **+++** |
| **28** | **-°** | **-** | **SCL** | **-°** | **-** | **-** | **<<SCL** | **-** | **<OL** | **<OL** | **<<OL°** | **<<OL°** | **-** | **-** | **-** | **-** |
| **29** | **<<OL°** | **-** | **+++** | **-** | **-** | **-** | **+++** | **+++** | **-** | **-** | **-** | **+++** | **-** | **-** | **+++** | **+++** |
| **30** | **++/-** | **<OL** | **+++** | **+++** | **+++** | **-** | **+++** | **-°** | **-** | **-** | **<<OL** | **<<OL** | **-** | **-** | **++/-** | **<OL** |
| **31** | **-** | **-** | **<SCL** | **-** | **-** | **<SCL** | **<SCL** | **-** | **-** | **-** | **P -** | **-** | **CL** | **-** | **-** | **-** |
| **32** | **-** | **-** | **<SCL** | **-** | **-** | **SCL** | **SCL** | **-°** | **OL** | **OL** | **P -** | **-** | **CL** | **-** | **-** | **-** |
| **33** | **-** | **-** | **<<SCL** | **-** | **-** | **-** | **<<SCL** | **-** | **-** | **-** | **P -** | **-** | **-** | **-** | **-** | **-** |
| **34** | **SCL** | **CL** | **+++** | **-** | **-** | **+++** | **+++** | **CL** | **<OL** | **<OL** | **CL** | **CL** | **<CL** | **-** | **CL** | **CL** |
| **35** | **CL** | **-°** | **<SCL** | **SCL** | **+++** | **-** | **+++** | **CL** | **-** | **-** | **CL** | **CL** | **-** | **-** | **<CL** | **-/++** |
| **36** | **<CL** | **-** | **<SCL** | **<<SCL** | **+++** | **-** | **+++** | **CL** | **<OL** | **<OL** | **CL** | **CL** | **-** | **-** | **CL** | **-°** |
| **37** | **CL** | **<<OL** | **SCL** | **+++** | **-/+** | **-** | **+++** | **CL** | **-** | **-** | **CL** | **CL** | **-** | **-** | **CL** | **<<OL** |
| **38** | **-** | **-** | **-** | **-** | **-** | **-** | **-** | **-** | **OL** | **OL** | **-** | **-** | **-** | **-** | **-** | **-** |
| **39** | **<<OL** | **-** | **+++** | **-** | **-** | **+++** | **+++** | **<OL** | **<OL** | **<OL** | **<<OL** | **<<OL** | **CL** | **-** | **<<OL** | **-** |
| **40** | **<OL** | **-** | **+++** | **-** | **-** | **-** | **+++** | **CL** | **OL** | **OL** | **CL** | **CL** | **-** | **-** | **SCL** | **-°** |
| **41** | **<CL** | **CL** | **<<SCL** | **<<SCL** | **+++** | **-** | **-** | **CL** | **OL** | **OL** | **CL** | **CL** | **-** | **+++** | **CL** | **<CL** |
| **42** | **<SCL** | **<<OL** | **<<SCL** | **-** | **-** | **-** | **+++** | **CL** | **<OL** | **<OL** | **SCL** | **SCL** | **-** | **-** | **<<SCL** |  |
| **43** | **<<OL** | **-** | **SCL** | **-** | **-** | **<<SCL** | **+++** | **<<OL** | **-** | **-** | **<<OL** | **<<OL** | **CL** | **-** | **<<OL** |  |
| **44** | **-°** | **<<OL** | **<<SCL** | **+++** | **+++** | **-** | **+++** | **P -** | **-** | **-** | **P -** | **-** | **-** | **-** | **P -** | **<SCL** |
| **45** | **<CL** | **<OL** | **+++** | **-** | **-/+** | **-** | **+++** | **CL** | **OL** | **OL** | **CL** | **CL** | **-** | **-/+** | **SCL** | **+++** |
| **46** | **-** | **-** | **<SCL** | **-** | **-** | **-** | **-** | **-** | **<OL** | **<OL** | **-** | **-** | **-** | **-** | **-** | **-** |
| **47** | **<CL** | **-** | **SCL** | **+++** | **+++** | **-** | **+++** | **CL** | **OL** | **OL** | **CL** | **CL** | **-** | **+++** | **CL** | **-** |
| **48** | **<CL** | **CL** | **SCL** | **<SCL** | **<<SCL** | **+++** | **-** | **CL** | **OL** | **OL** | **CL** | **CL** | **SCL** | **SCL** | **CL** | **SCL** |
| **49** | **<CL** | **-** | **SCL** | **<<SCL** | **<<SCL** | **<SCL** | **<CL** | **CL** | **OL** | **OL** | **CL** | **CL** | **CL** | **CL** | **CL** | **-** |
| **50** | **SCL** | **-** | **SCL** | **-** | **-** | **<<SCL** | **+++** | **CL** | **OL** | **-** | **CL** | **CL** | **CL** | **-** | **<OL** | **-** |
| **51** | **-** | **-** | **+++** | **-** | **-** | **+++** | **<<SCL** | **-** | **<OL** | **-** | **-/+** | **-** | **+++** | **-** | **-** | **-** |
| **52** | **SCL** | **-** | **+++** | **+** | **-** | **-** | **<<SCL** | **SCL** | **-** | **-** | **<CL** | **<<OL** | **-** | **++** | **<<SCL** | **-** |
| **53** | **<<OL** | **-** | **<<SCL** | **+++** | **+++** | **+++** | **<<SCL** | **<<OL** | **+++** | **<<OL** | **<<OL** | **<<OL** | **<<SCL** | **-** | **+++** | **+++** |
| **54** | **<CL** | **CL** | **+++** | **-** | **-** | **+++** | **++** | **<CL** | **-** | **-** | **<CL** | **SCL** | **<<SCL** | **-** | **<<SCL** | **<SCL** |
| **55** | **<CL** | **CL** | **<<SCL** | **<<SCL** | **+++** | **-** | **-** | **CL** | **-** | **-** | **CL** | **CL** | **SCL** | **SCL** | **CL** | **SCL** |
| **56** | **<CL** | **CL** | **+++** | **<<SCL** | **+++** | **-** | **-** | **CL** | **-** | **-** | **CL** | **CL** | **-** | **+++** | **CL** | **SCL** |
| **57** | **<<OL** | **<<OL** | **<<SCL** | **+++** | **+++** | **-** | **+++** | **<<OL** | **<OL** | **OL** | **-** | **-** | **-** | **-** | **+** | **SCL** |
| **58** | **-** | **<<OL** | **<<SCL** | **+** | **+** | **-** | **+++** | **+** | **-** | **-** | **-** | **-** | **-** | **-** | **-** | **SCL** |
| **59** | **-** | **+** | **<<SCL** | **+++** | **+++** | **+++** | **<<SCL** | **+** | **+++** | **<<OL** | **P -** | **P -** | **SCL** | **+++** | **-** | **<<SCL** |
| **60** | **-** | **+** | **+++** | **+++** | **+++** | **-** | **+++** | **-** | **-** | **-** | **P -** | **-** | **-** | **-** | **-** | **<SCL** |
| **61** | **SCL** | **<<OL** | **<<SCL** | **+++** | **+++** | **+++** | **-** | **CL** | **-** | **-** | **CL** | **CL** | **SCL** | **+++** | **SCL** | **+++** |
| **62** | **<CL** | **-** | **<<SCL** | **+++** | **++** | **-** | **<<SCL** | **-** | **+++** | **<OL** | **P -** | **-** | **-** | **+++** | **<CL** | **-** |
| **63** | **CL** | **SCL** | **-** | **-** | **-** | **-** | **SCL** | **CL** | **-** | **-** | **CL** | **CL** | **-** | **-** | **CL** | **SCL** |
| **64 (prov)** | **-** | **-** | **<<OL** | **+++** | **+++** | **-** | **+++** | **-** | **-** | **-** | **-** | **-** | **-** | **+++** | **-** | **<<SCL** |
| **65 (prov)** | **-** | **-** | **+++** | **<<SCL** | **+++** | **+++** | **<<SCL** | **-** | **<OL** | **<OL** | **P -** | **-** | **+++** | **-** | **-** | **+++** |
| **66 (prov)** | **-** | **-** | **<<SCL** | **-** | **-** | **+++** | **SCL** | **<<OL** | **<OL** | **<OL** | **P -** | **-** | **+++** | **-** | **-** | **-** |
| **67** | **CL** | **CL** | **<<SCL** | **-** | **-** | **-** | **-** | **CL** | **-** | **-** | **CL** | **CL** | **-** | **-** | **CL** | **<<SCL** |
| **68** | **CL** | **-** | **SCL** | **<<SCL** | **-** | **<<SCL** | **<<SCL** | **CL** | **OL** | **OL** | **CL** | **CL** | **<<SCL** | **-** | **<CL** | **-** |
| **69 (prov)** | **+++** | **-** | **+++** | **-** | **-** | **-** | **+++** | **-** | **-** | **-** | **-** | **-** | **-** | **-** | **-** | **+++** |
| **70 (prov)** | **CL** | **<<SCL** | **-** | **-** | **-** | **-** | **<<OL** | **CL** | **<<OL** | **<OL** | **CL** | **CL** | **-** | **-** | **CL** | **+++** |
| **71 (prov)** | **CL** | **-** | **+++** | **-** | **-** | **+++** | **<<SCL** | **CL** | **-** | **-** | **CL** | **CL** | **+++** | **-** | **<CL** | **+++** |
| **72 (prov)** | **-** | **-** | **+++** | **-** | **-** | **+++** | **-** | **-** | **<OL** | **<OL** | **-** | **-** | **++** | **-** | **-** | **-** |
| **73 (Not yet)** |  |  |  |  |  |  |  |  |  |  |  |  |  |  |  |  |
| **74 (prov)** | **CL** | **<<SCL** | **-** | **-** | **-** | **-** | **-** | **CL** | **<OL** | **OL** | **CL** | **<CL** | **-** | **-** | **SCL** | **SCL** |
| **75 (prov)** | **<OL** | **-** | **+++** | **<OL** | **+++** | **+++** | **-** | **CL** | **-** | **-** | **CL** | **SCL** | **+++** | **<<OL** | **+++** | **-** |
| **76 (prov)** | **<<OL** | **-** | **<<SCL** | **+++** | **-** | **<<SCL** | **SCL** | **<<OL** | **<OL** | **OL** | **<<OL** | **<<OL** | **+++** | **-** | **-** | **-** |
| **77 (Not yet)** |  |  |  |  |  |  |  |  |  |  |  |  |  |  |  |  |
| **78 (prov)** | **<<OL** | **-** | **<<SCL** | **-** | **-** | **-** | **<<SCL** | **<<OL** | **<OL** | **OL** | **<<OL** | **<<OL** | **-** | **-** | **-** | **-** |
| **79 (prov)** | **<<OL** | **-** | **-** | **-** | **-** | **-** | **<<SCL** | **<<OL** | **<OL** | **OL** | **<<OL** | **<<OL** | **-** | **-** | **<<OL** | **-** |
| **80 (prov)** | **<<OL** | **-** | **-** | **-** | **-** | **-** | **<<SCL** | **<<OL** | **<OL** |  | **<<OL** | **<<OL** | **-** | **-** | **-** | **-** |
| **81** | **<<OL** | **-** | **-** | **-** | **-** | **-** | **-** | **<<OL** | **<OL** | **<OL** | **<<OL** | **<<OL** | **-** | **-** | **<<OL** | **-** |
| **82** | **-** | **-** | **<<SCL** | **-** | **-** | **+++** | **<<SCL** | **CL** | **-** | **-** | **<CL** | **<<SCL** | **+++** | **-** | **-** | **-** |
| **88** | **+++** | **++** | **SCL** | **-** | **SCL** | **-** | **CL** | **SCL** | **-** | **-** | **+++** | **+++** | **-** | **-** | **+++** | **SCL** |
| **Untypable** | **-** | **-** | **-** | **-** | **-** | **-** | **-** | **-** | **-** | **-** | **-** | **-** | **-** | **-** | **-** | **-** |

**Table 3.** Table detailing the accessory variation of Group 1 as depicted in Figure 4. Presence of accessory gene in phage genome depicted by X and absence by blank box.

| **Group 1** | **Phage 1** | **Phage 8** | **Phage 11** | **Phage 12** | **Phage 15** |
| --- | --- | --- | --- | --- | --- |
| **PROKKA1_00033_tRNA-Arg(tct)** | X |  |  |  | X |
| **PROKKA1_00042_tRNA-Pro(tgg)** | X | X | X | X |  |
| **PROKKA1_00078_hypothetical_protein** | X |  |  |  | X |
| **PROKKA1_00081_hypothetical_protein** | X |  |  |  | X |
| **PROKKA1_00083_hypothetical_protein** | X |  |  |  | X |
| **PROKKA1_00092_hypothetical_protein** | X |  |  |  | X |
| **PROKKA1_00094_hypothetical_protein** | X |  |  |  | X |
| **PROKKA1_00130_hypothetical_protein** | X | X | X | X |  |
| **PROKKA8_00001_hypothetical_protein** |  | X |  |  |  |
| **PROKKA8_00007_AP2_domain_protein** |  | X | X | X |  |
| **PROKKA8_00039_hypothetical_protein** |  | X | X | X |  |
| **PROKKA8_00042_hypothetical_protein** |  | X | X | X |  |
| **PROKKA8_00087_tRNA-Trp(cca)** |  | X | X | X | X |
| **PROKKA8_00088_hypothetical_protein** |  | X | X | X | X |
| **PROKKA15_00062_tRNA-Pro(tgg)** |  |  |  |  | X |
| **PROKKA15_00145_hypothetical_protein** |  |  |  |  | X |
|  |  |  |  |  |  |

**Table 4.** Table detailing the accessory variation of Group 2 as depicted in Figure 5. Presence of accessory gene in phage genome depicted by X and absence by blank box.

| **Group 2** | **Phage 3** | **Phage 6** | **Phage 7** | **Phage 13** |
| --- | --- | --- | --- | --- |
| **PROKKA7_00005_Phage_tail_fibre_adhesin_Gp38** |  |  | X |  |
| **PROKKA7_00008_hypothetical_protein** |  |  | X |  |
| **PROKKA3_00008_hypothetical_protein** | X | X |  | X |
| **PROKKA7_00009_hypothetical_protein** |  |  | X |  |
| **PROKKA7_00010_hypothetical_protein** |  |  | X |  |
| **PROKKA7_00011_hypothetical_protein** |  |  | X |  |
| **PROKKA3_00011_hypothetical_protein** | X | X |  | X |
| **PROKKA7_00015_hypothetical_protein** |  |  | X |  |
| **PROKKA6_00016_hypothetical_protein** | X | X |  | X |
| **PROKKA3_00016_hypothetical_protein** | X | X |  | X |
| **PROKKA7_00017_hypothetical_protein** |  |  | X |  |
| **PROKKA7_00019_hypothetical_protein** |  |  | X |  |
| **PROKKA7_00020_hypothetical_protein** |  |  | X |  |
| **PROKKA7_00021_hypothetical_protein** |  |  | X |  |
| **PROKKA3_00021_hypothetical_protein** | X | X |  | X |
| **PROKKA7_00022_hypothetical_protein** |  |  | X |  |
| **PROKKA7_00023_hypothetical_protein** |  |  | X |  |
| **PROKKA3_00023_hypothetical_protein** | X | X |  | X |
| **PROKKA3_00024_hypothetical_protein** | X | X |  | X |
| **PROKKA7_00026_hypothetical_protein** |  |  | X |  |
| **PROKKA7_00027_hypothetical_protein** |  |  | X |  |
| **PROKKA3_00028_Phospho-2-dehydro-3-deoxyheptonate_aldolase_Tyr-sensitive** | X | X |  | X |
| **PROKKA3_00030_hypothetical_protein** | X | X |  | X |
| **PROKKA7_00032_hypothetical_protein** |  |  | X |  |
| **PROKKA7_00033_hypothetical_protein** |  |  | X |  |
| **PROKKA3_00034_Phage_GP30.8_protein** | X | X |  | X |
| **PROKKA3_00036_hypothetical_protein** | X | X |  | X |
| **PROKKA7_00037_hypothetical_protein** |  |  | X |  |
| **PROKKA7_00038_hypothetical_protein** |  |  | X |  |
| **PROKKA3_00038_hypothetical_protein** | X | X |  | X |
| **PROKKA7_00040_hypothetical_protein** |  |  | X |  |
| **PROKKA3_00041_hypothetical_protein** | X | X |  | X |
| **PROKKA7_00042_hypothetical_protein** |  |  | X |  |
| **PROKKA7_00043_hypothetical_protein** |  |  | X |  |
| **PROKKA7_00044_hypothetical_protein** |  |  | X |  |
| **PROKKA3_00045_hypothetical_protein** | X | X |  | X |
| **PROKKA7_00048_hypothetical_protein** |  |  | X |  |
| **PROKKA7_00049_hypothetical_protein** |  |  | X |  |
| **PROKKA7_00050_hypothetical_protein** |  |  | X |  |
| **PROKKA7_00051_hypothetical_protein** |  |  | X |  |
| **PROKKA7_00052_hypothetical_protein** |  |  | X |  |
| **PROKKA7_00053_hypothetical_protein** |  |  | X |  |
| **PROKKA7_00054_hypothetical_protein** |  |  | X |  |
| **PROKKA7_00055_hypothetical_protein** |  |  | X |  |
| **PROKKA7_00056_hypothetical_protein** |  |  | X |  |
| **PROKKA7_00058_hypothetical_protein** |  |  | X |  |
| **PROKKA7_00060_hypothetical_protein** |  |  | X |  |
| **PROKKA3_00062_hypothetical_protein** | X | X |  | X |
| **PROKKA3_00065_hypothetical_protein** | X | X |  | X |
| **PROKKA7_00066_hypothetical_protein** |  |  | X |  |
| **PROKKA7_00071_hypothetical_protein** |  |  | X |  |
| **PROKKA7_00072_Thymidylate_synthase_1** |  |  | X |  |
| **PROKKA7_00073_hypothetical_protein** |  |  | X |  |
| **PROKKA7_00074_hypothetical_protein** |  |  | X |  |
| **PROKKA7_00075_hypothetical_protein** |  |  | X |  |
| **PROKKA7_00077_hypothetical_protein** |  |  | X |  |
| **PROKKA7_00082_Phage_RNA_polymerase_binding_RpbA** |  |  | X |  |
| **PROKKA7_00085_hypothetical_protein** |  |  | X |  |
| **PROKKA7_00086_hypothetical_protein** |  |  | X |  |
| **PROKKA7_00088_DNA_alpha-glucosyltransferase** |  |  | X |  |
| **PROKKA7_00091_hypothetical_protein** |  |  | X |  |
| **PROKKA7_00093_hypothetical_protein** |  |  | X |  |
| **PROKKA3_00097_Nuclease_inhibitor_from_bacteriophage_T4** | X | X |  | X |
| **PROKKA3_00098_hypothetical_protein** | X | X |  |  |
| **PROKKA3_00099_hypothetical_protein** | X |  |  |  |
| **PROKKA7_00099_hypothetical_protein** |  |  | X |  |
| **PROKKA3_00100_hypothetical_protein** | X |  |  |  |
| **PROKKA3_00101_hypothetical_protein** | X |  |  |  |
| **PROKKA3_00102_hypothetical_protein** | X |  |  |  |
| **PROKKA3_00103_tRNA-Met(cat)** | X |  |  |  |
| **PROKKA3_00104_tRNA-Arg(tct)** | X |  |  |  |
| **PROKKA3_00105_hypothetical_protein** | X |  |  |  |
| **PROKKA3_00106_hypothetical_protein** | X |  |  |  |
| **PROKKA3_00107_hypothetical_protein** | X |  |  |  |
| **PROKKA7_00107_hypothetical_protein** |  |  | X |  |
| **PROKKA3_00108_hypothetical_protein** | X |  |  |  |
| **PROKKA3_00109_Bacteriophage_FRD3_protein** | X |  |  |  |
| **PROKKA7_00109_hypothetical_protein** |  |  | X |  |
| **PROKKA3_00110_hypothetical_protein** | X |  |  |  |
| **PROKKA3_00111_hypothetical_protein** | X |  |  |  |
| **PROKKA7_00111_hypothetical_protein** |  |  | X |  |
| **PROKKA3_00112_hypothetical_protein** | X |  |  |  |
| **PROKKA7_00112_hypothetical_protein** |  |  | X |  |
| **PROKKA3_00113_hypothetical_protein** | X |  |  |  |
| **PROKKA3_00114_hypothetical_protein** | X |  |  |  |
| **PROKKA7_00114_hypothetical_protein** |  |  | X |  |
| **PROKKA3_00115_hypothetical_protein** | X |  |  |  |
| **PROKKA7_00115_hypothetical_protein** |  |  | X |  |
| **PROKKA3_00116_hypothetical_protein** | X |  |  |  |
| **PROKKA7_00116_hypothetical_protein** |  |  | X |  |
| **PROKKA7_00117_hypothetical_protein** |  |  | X |  |
| **PROKKA3_00117_hypothetical_protein** | X |  |  | X |
| **PROKKA3_00118_hypothetical_protein** | X |  |  |  |
| **PROKKA3_00119_hypothetical_protein** | X |  |  | X |
| **PROKKA7_00119_hypothetical_protein** |  |  | X |  |
| **PROKKA3_00120_hypothetical_protein** | X | X |  | X |
| **PROKKA3_00121_hypothetical_protein** | X | X |  | X |
| **PROKKA3_00122_hypothetical_protein** | X | X |  | X |
| **PROKKA3_00123_hypothetical_protein** | X | X |  |  |
| **PROKKA7_00123_hypothetical_protein** |  |  | X |  |
| **PROKKA7_00125_hypothetical_protein** |  |  | X |  |
| **PROKKA7_00126_hypothetical_protein** |  |  | X |  |
| **PROKKA3_00126_hypothetical_protein** | X | X |  | X |
| **PROKKA3_00128_hypothetical_protein** | X | X |  |  |
| **PROKKA7_00128_hypothetical_protein** |  |  | X |  |
| **PROKKA7_00129_hypothetical_protein** |  |  | X |  |
| **PROKKA7_00130_hypothetical_protein** |  |  | X |  |
| **PROKKA7_00131_hypothetical_protein** |  |  | X |  |
| **PROKKA7_00132_hypothetical_protein** |  |  | X |  |
| **PROKKA7_00136_hypothetical_protein** |  |  | X |  |
| **PROKKA7_00137_hypothetical_protein** |  |  | X |  |
| **PROKKA7_00138_hypothetical_protein** |  |  | X |  |
| **PROKKA3_00141_hypothetical_protein** | X | X |  | X |
| **PROKKA3_00145_hypothetical_protein** | X | X |  | X |
| **PROKKA3_00146_hypothetical_protein** | X | X |  | X |
| **PROKKA3_00148_hypothetical_protein** | X | X |  | X |
| **PROKKA7_00151_hypothetical_protein** |  |  | X |  |
| **PROKKA3_00152_hypothetical_protein** | X | X |  | X |
| **PROKKA3_00154_hypothetical_protein** | X | X |  | X |
| **PROKKA7_00155_hypothetical_protein** |  |  | X |  |
| **PROKKA3_00156_hypothetical_protein** | X | X |  | X |
| **PROKKA7_00156_hypothetical_protein** |  |  | X |  |
| **PROKKA7_00157_hypothetical_protein** |  |  | X |  |
| **PROKKA3_00157_hypothetical_protein** | X | X |  | X |
| **PROKKA3_00159_hypothetical_protein** | X | X |  | X |
| **PROKKA3_00160_hypothetical_protein** | X | X |  | X |
| **PROKKA7_00160_hypothetical_protein** |  |  | X |  |
| **PROKKA7_00161_hypothetical_protein** |  |  | X |  |
| **PROKKA3_00163_hypothetical_protein** | X | X |  | X |
| **PROKKA7_00164_tRNA-Arg(tct)** |  |  | X |  |
| **PROKKA3_00165_hypothetical_protein** | X | X |  | X |
| **PROKKA7_00165_tRNA-His(gtg)** |  |  | X |  |
| **PROKKA3_00166_hypothetical_protein** | X | X |  | X |
| **PROKKA7_00166_tRNA-Asn(gtt)** |  |  | X |  |
| **PROKKA7_00167_tRNA-Tyr(gta)** |  |  | X |  |
| **PROKKA7_00168_tRNA-Met(cat)** |  |  | X |  |
| **PROKKA7_00169_tRNA-Thr(tgt)** |  |  | X |  |
| **PROKKA7_00170_tRNA-Ser(tga)** |  |  | X |  |
| **PROKKA7_00171_tRNA-Pro(tgg)** |  |  | X |  |
| **PROKKA7_00172_tRNA-Gly(tcc)** |  |  | X |  |
| **PROKKA7_00173_tRNA-Leu(taa)** |  |  | X |  |
| **PROKKA3_00173_hypothetical_protein** | X | X |  | X |
| **PROKKA7_00174_tRNA-Gln(ttg)** |  |  | X |  |
| **PROKKA3_00174_hypothetical_protein** | X | X |  | X |
| **PROKKA7_00176_hypothetical_protein** |  |  | X |  |
| **PROKKA3_00178_hypothetical_protein** | X | X |  | X |
| **PROKKA7_00178_hypothetical_protein** |  |  | X |  |
| **PROKKA3_00181_hypothetical_protein** | X | X |  | X |
| **PROKKA3_00183_hypothetical_protein** | X | X |  | X |
| **PROKKA3_00186_hypothetical_protein** | X | X |  | X |
| **PROKKA3_00188_hypothetical_protein** | X | X |  | X |
| **PROKKA3_00189_hypothetical_protein** | X | X |  | X |
| **PROKKA3_00192_Phage_RNA_polymerase_binding_RpbA** | X | X |  | X |
| **PROKKA3_00198_Arabinose_5-phosphate_isomerase_KpsF** | X | X |  | X |
| **PROKKA3_00199_hypothetical_protein** | X | X |  | X |
| **PROKKA3_00200_CTP:phosphocholine_cytidylyltransferase_involved_in_choline_phosphorylation_for_cell_surface_LPS_epitopes** | X | X |  | X |
| **PROKKA3_00201_capsule_biosynthesis_phosphatase** | X | X |  | X |
| **PROKKA3_00202_Collagenase** | X | X |  | X |
| **PROKKA3_00203_hypothetical_protein** | X | X |  | X |
| **PROKKA3_00204_Thymidylate_synthase** | X | X |  | X |
| **PROKKA3_00205_hypothetical_protein** | X | X |  | X |
| **PROKKA3_00206_hypothetical_protein** | X | X |  | X |
| **PROKKA3_00210_hypothetical_protein** | X | X |  | X |
| **PROKKA3_00211_hypothetical_protein** | X | X |  | X |
| **PROKKA7_00212_hypothetical_protein** |  |  | X |  |
| **PROKKA3_00213_hypothetical_protein** | X | X |  | X |
| **PROKKA3_00214_hypothetical_protein** | X | X |  | X |
| **PROKKA3_00217_hypothetical_protein** | X | X |  | X |
| **PROKKA3_00220_hypothetical_protein** | X | X |  | X |
| **PROKKA3_00222_hypothetical_protein** | X | X |  | X |
| **PROKKA3_00223_hypothetical_protein** | X | X |  | X |
| **PROKKA3_00227_hypothetical_protein** | X | X |  | X |
| **PROKKA3_00228_hypothetical_protein** | X | X |  | X |
| **PROKKA3_00229_hypothetical_protein** | X | X |  | X |
| **PROKKA7_00229_hypothetical_protein** |  |  | X |  |
| **PROKKA7_00230_ADP-ribosyltransferase_exoenzyme** |  |  | X |  |
| **PROKKA3_00230_hypothetical_protein** | X | X |  | X |
| **PROKKA3_00231_hypothetical_protein** | X | X |  | X |
| **PROKKA3_00232_hypothetical_protein** | X | X |  | X |
| **PROKKA7_00234_hypothetical_protein** |  |  | X |  |
| **PROKKA7_00237_hypothetical_protein** |  |  | X |  |
| **PROKKA3_00238_hypothetical_protein** | X | X |  | X |
| **PROKKA3_00239_hypothetical_protein** | X | X |  | X |
| **PROKKA7_00239_hypothetical_protein** |  |  | X |  |
| **PROKKA3_00240_hypothetical_protein** | X | X |  | X |
| **PROKKA7_00241_Phage_GP30.8_protein** |  |  | X |  |
| **PROKKA3_00244_hypothetical_protein** | X | X |  |  |
| **PROKKA7_00245_hypothetical_protein** |  |  | X |  |
| **PROKKA7_00246_hypothetical_protein** |  |  | X |  |
| **PROKKA3_00246_hypothetical_protein** | X | X |  | X |
| **PROKKA7_00248_hypothetical_protein** |  |  | X |  |
| **PROKKA3_00250_hypothetical_protein** | X | X |  | X |
| **PROKKA7_00251_hypothetical_protein** |  |  | X |  |
| **PROKKA7_00252_hypothetical_protein** |  |  | X |  |
| **PROKKA3_00253_hypothetical_protein** | X | X |  | X |
| **PROKKA3_00254_hypothetical_protein** | X | X |  | X |
| **PROKKA3_00256_hypothetical_protein** | X | X |  | X |
| **PROKKA7_00258_hypothetical_protein** |  |  | X |  |
| **PROKKA3_00261_hypothetical_protein** | X | X |  | X |
| **PROKKA3_00262_hypothetical_protein** | X | X |  | X |
| **PROKKA3_00263_hypothetical_protein** | X | X |  | X |
| **PROKKA7_00263_hypothetical_protein** |  |  | X |  |
| **PROKKA3_00264_hypothetical_protein** | X | X |  | X |
| **PROKKA7_00266_hypothetical_protein** |  |  | X |  |
| **PROKKA3_00267_Caudovirales_tail_fibre_assembly_protein** | X | X |  | X |
| **PROKKA7_00268_hypothetical_protein** |  |  | X |  |
| **PROKKA3_00268_Phage_Tail_Collar_Domain_protein** | X | X |  |  |
| **PROKKA7_00269_hypothetical_protein** |  |  | X |  |
| **PROKKA7_00271_hypothetical_protein** |  |  | X |  |
| **PROKKA7_00273_Bacteriophage_FRD3_protein** |  |  | X |  |
| **PROKKA7_00279_hypothetical_protein** |  |  | X |  |
| **PROKKA3_00277_Bacteriophage_replication_gene_A_protein_(GPA)** | X | X |  | X |
| **PROKKA7_00285_hypothetical_protein** |  |  | X |  |

**Table 5.** Table detailing the accessory variation of Group 3 as depicted in Figure 6. Presence of accessory gene in phage genome depicted by X and absence by blank box.

| **Group 3** | **Phage 4** | **Phage 5** | **Phage 14** |
| --- | --- | --- | --- |
| **PROKKA5_00002_hypothetical_protein** |  | X | X |
| **PROKKA5_00003_hypothetical_protein** |  | X | X |
| **PROKKA4_00012_AP2_domain_protein** | X |  |  |
| **PROKKA5_00016_hypothetical_protein** |  | X |  |
| **PROKKA4_00030_hypothetical_protein** | X |  |  |
| **PROKKA4_00064_hypothetical_protein** | X | X |  |
| **PROKKA4_00065_hypothetical_protein** | X |  |  |
| **PROKKA4_00079_hypothetical_protein** | X |  |  |
| **PROKKA14_00088_hypothetical_protein** |  |  | X |
| **PROKKA4_00104_Recombination_endonuclease_VII** | X |  | X |
| **PROKKA5_00106_(putative HNH homing endonuclease)** |  | X |  |
| **PROKKA5_00115_tRNA-???(atcc)** |  | X | X |
| **PROKKA5_00116_tRNA-Ser(tga)** |  | X | X |
| **PROKKA5_00117_tRNA-Tyr(gta)** |  | X | X |
| **PROKKA14_00139_(putative quinate dehydrogenase)** |  |  | X |
| **PROKKA14_00143_hypothetical_protein** |  |  | X |
| **PROKKA5_00159_(putative minor capsid protein)** |  | X |  |
| **PROKKA4_00164_hypothetical_protein** | X |  |  |
| **PROKKA4_00167_hypothetical_protein** | X |  |  |
| **PROKKA4_00168_hypothetical_protein** | X | X |  |
| **PROKKA5_00171_hypothetical_protein** |  | X | X |
| **PROKKA4_00198_hypothetical_protein** | X | X |  |
| **PROKKA4_00204_hypothetical_protein** | X |  |  |
| **PROKKA4_00205_hypothetical_protein** | X |  | X |
| **PROKKA5_00205_hypothetical_protein** | X | X |  |
| **PROKKA5_00207_hypothetical_protein** | X | X |  |
| **PROKKA5_00210_hypothetical_protein** |  | X |  |
| **PROKKA4_00217_(putative endonuclease VII)** | X | X |  |
| **PROKKA5_00228_(putative prophage MuMc02)** |  | X |  |

**Table 6**. Table representing unique reactions that only occur within a subset of groups 1, 2 and 3 with specific PTs and number of genes found only in that subset. The number of genes was determined looking at the accessory gene regions within each group and identify those found in a subset of the phages.

| **Group** | **Members** | **PTs that react with subsets of the group** | **No. of gene differences for that subset** |
| --- | --- | --- | --- |
| **1** | Single member of group 1 | TP8 reacts with PT58 TP11 reacts with PT6,7, 13,17, 21,31,32,33,51,60 TP12 reacts with PT16 TP15 reacts with no PTs | 1 0 0 2 |
|  | Two members of group 1 | TP11 and 12 react with PT5,18, 19,25,28 TP1 and 15 react with no PTs | 0 6 |
|  | Three members of group 1 | TP8, 11 and 12 react with PT21/28,59,82 TP1, 15 and 11 react with PT62 TP11, 15 and 8 react with PT44 TP1, 15 and 12 react with PT30 TP1, 15 and 8 react with PT57 | 5 0 0 0 0 |
|  | Four members of group 1 | TP1, 8, 11 and 12 react with PT9 TP1, 8, 15 and 12 react with PT29 TP1, 11, 12 and 15 react with PT3 TP8, 11, 12 and 15 react with no PTs | 2 0 0 2 |
|  | **All** | **1,2,4,8,10,11,12,14,15,23,24,26,27,34,35,36,37,39,40,41,45,47,48,49,50,52,53,54,55,56,61,63,67,68,81,88** |  |
|  | **None** | **20,38,46** |  |
| **2** | Single member of group 2 | TP3 reacts with PT3,7,11,40,41,56,67 TP7 reacts with PT19,63 | 19 107 |
|  | Two members of group 2 | TP3 and 6 react with no PTs TP3 and 7 react with PT1,4,21,21/28,23,24,26,28,29,30,33,35,36,37,44,45,47,52,57,58,60,62,88  PT3 and 13 react with PT55 | 5 0   2 |
|  | Three members of group 2 | TP3, 6 and 7 react with PT9 TP3, 7 and 13 react with PT12 TP6, 7 and 13 react with PT46 TP3, 6 and 14 react with PT20,48, 61 | 0 0 0 80 |
|  | **All** | **2,5,6,8,13,14,15,16,17,18,25,27,31,32,34,39,49,50,51,53,54,59,68,82** |  |
|  | **None** | **10,38,81** |  |
| **3** | Single member of group 3 | TP4 reacts with PT2,5,6,13,15,16,17,18, 20,25,27,68 TP5 reacts with PT88 TP14 reacts with no PTs | 7 5  3 |
|  | Two members of group 3 | TP4 and 5 react with PT12,26,35,36,37,44,53,57,58,60 TP4 and 14 react with PT52 TP5 and 14 react with PT45 | 4 2 8 |
|  | **All** | **1,4,7,8,10,11,14,41,47,48,49,55,56,59,61,62** |  |
|  | **None** | **3,19,21,21/28,23,24,28,29,31,32,33,34,38,39,40,46,50,51,54,63,67,81,82** |  |


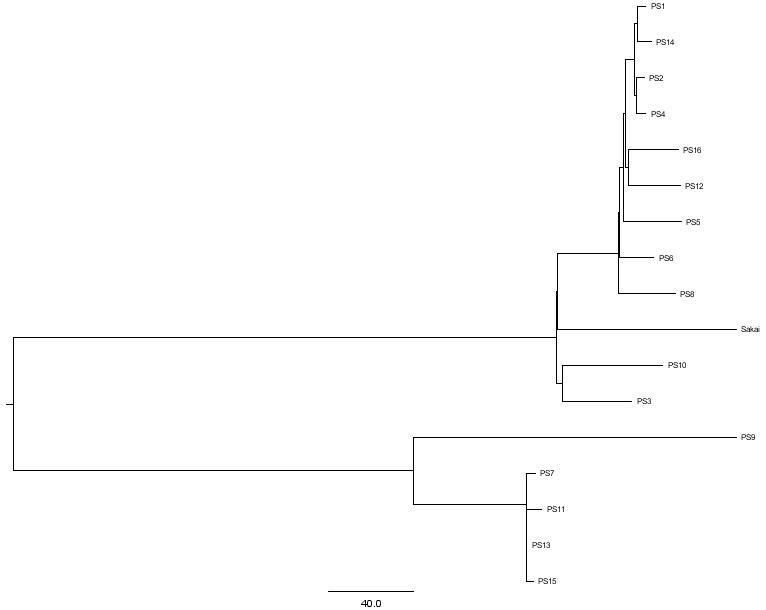


**Figure 1.** Phylogenetic tree of propagating strains for each typing phage and sakai as a reference. Snps were called using a mapping technique against sakai and tree drawn using MEGA 5.2. The labels stand for Propagating strain and then the corresponding phage number that they propagate. The data for this tree has been deposited in TreeBase with the submission ID 17186.


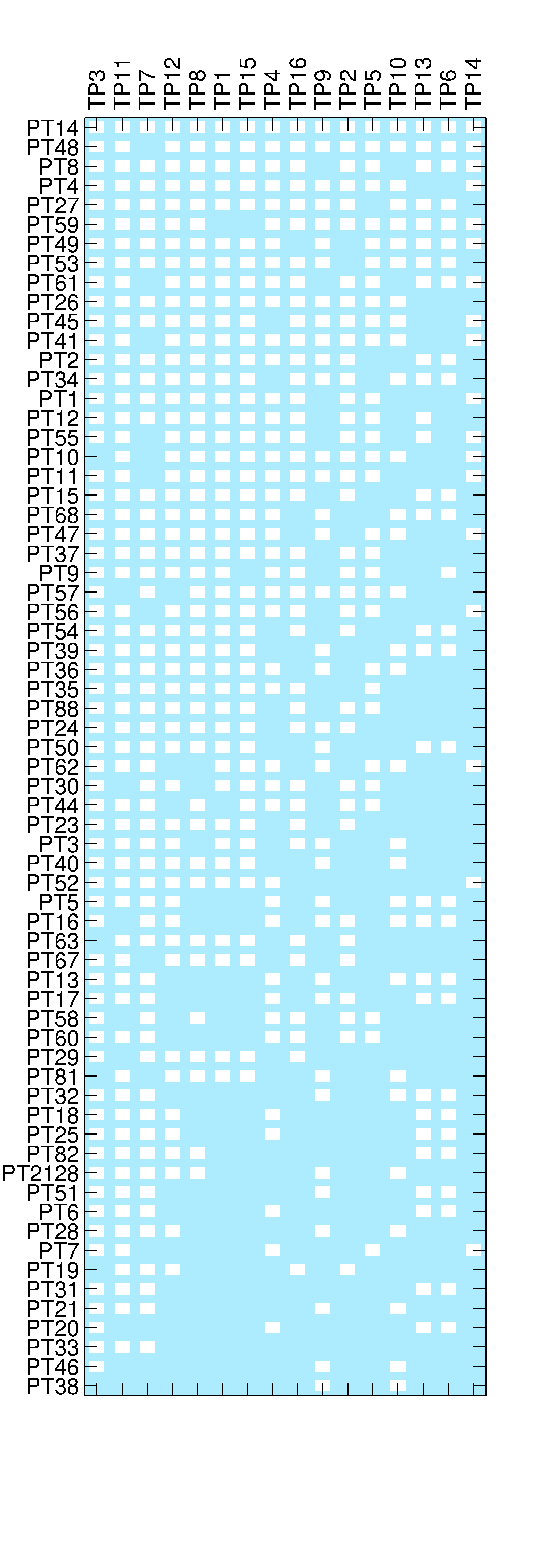


**Figure 2.** A visual representation of the degree of nestedness found within the classified *Escherichia coli O157:H7* phage-bacteria infection network. Each square represents an association between the corresponding phage and bacterial strains. Typing phages (TP) are ranked from highest to lowest phage infectivity range, whilst bacterial phage types (PT) are ordered from lowest to highest host resistance range.


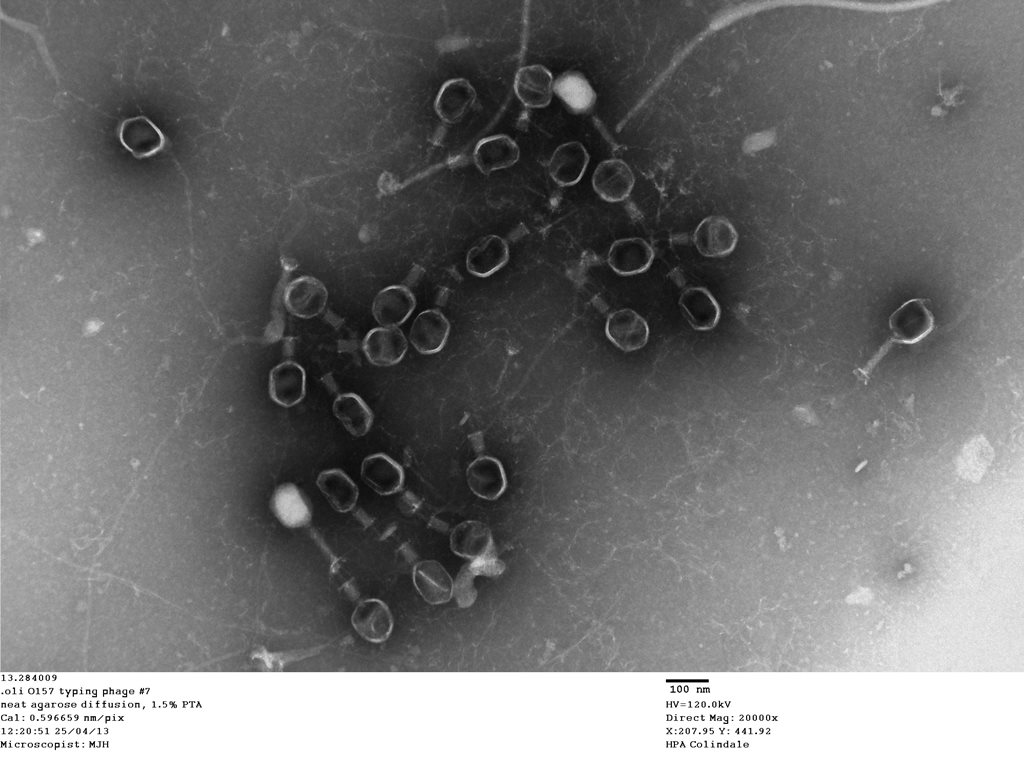


**Figure 3.** Electron Microscopy image of typing phage 7, a representation of T4-like long-tailed phage morphology.


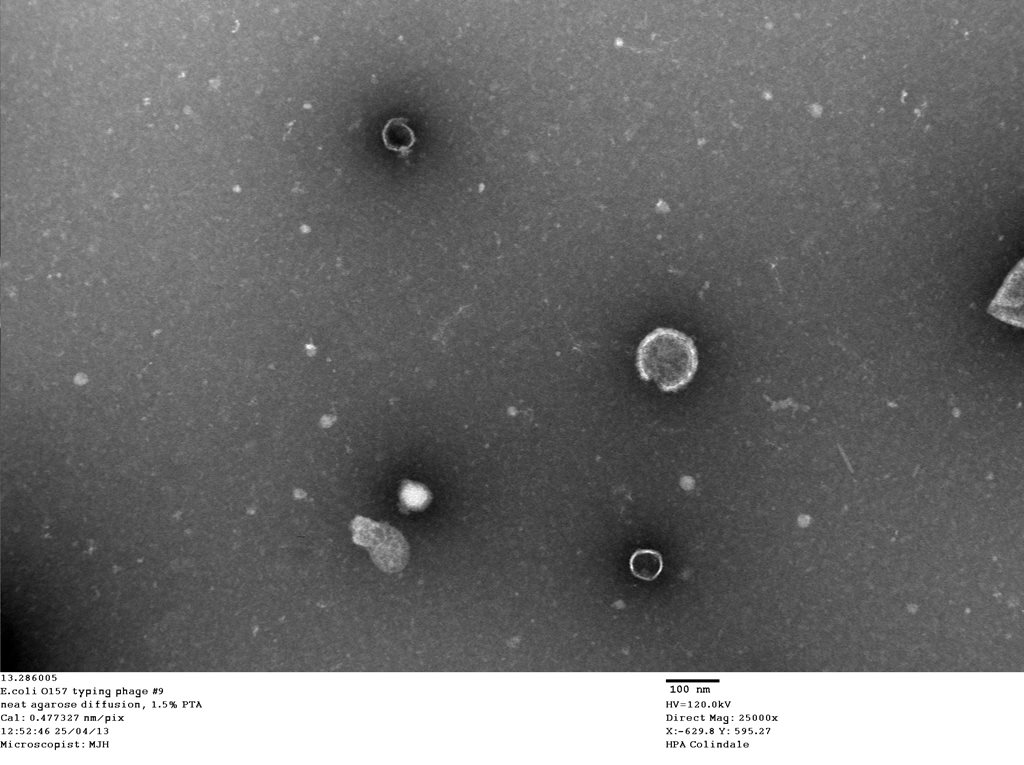


**Figure 4.** Electron Microscopy image of typing phage 9, a representation of T7-like short-tailed phage morphology. This image shows two phages and one is indicated by a red arrow.
